# Understanding the Diagnosis of Primary Headaches in Outpatients Referred to Japanese Headache Specialists

**DOI:** 10.7759/cureus.100122

**Published:** 2025-12-26

**Authors:** Yuki Tatsuno, Katsuya Takeuchi, Daisuke Danno, Shoji Kikui, Yumi Kawata, Takao Takeshima

**Affiliations:** 1 Medical Affairs, Hedgehog MedTech, Inc, Tokyo, JPN; 2 Headache Center, Department of Neurology, Tominaga Hospital, Osaka, JPN

**Keywords:** diagnostic accuracy, misdiagnosis, outpatients, primary headache disorders, underdiagnosis

## Abstract

Background

The timely and accurate diagnosis of primary headaches is crucial for enhancing patients’ quality of life. However, primary headaches have been reported to be misdiagnosed and underdiagnosed worldwide, especially by nonspecialists. In Japan, despite the high prevalence of primary headaches, research on the extent of headache misdiagnosis and underdiagnosis remains limited. Therefore, we aimed to assess the rates of concordant primary headache diagnoses between previous physicians and headache specialists among outpatients who were referred to the headache center in Japan.

Methodology

In this retrospective, observational study, we collected data at the renowned Headache Center of Tominaga Hospital in Japan from August 2024 through April 2025. The target population was headache patients aged six years or older who were referred from or previously visited another medical institution. We classified headache diagnoses into the following five categories: migraine; tension-type headache; trigeminal autonomic cephalalgia; other primary headaches; and other headaches, including secondary headaches and painful cranial neuropathies. We calculated the accuracy rate of previous physicians’ diagnoses by comparing theirs with those made by board-certified headache specialists at Tominaga Hospital.

Results

We analyzed 147 patients (mean age = 41.5 years; SD = 18.0 years) who were referred to Tominaga Hospital for headaches. Of these patients, 47 (32.0%) were male, and 100 (68.0%) were female. Overall, 69 (47.3%) patients were undiagnosed by previous physicians. The overall accuracy rate of previous physicians’ diagnoses was 43.8% (95% confidence interval (CI) = 36.0-51.9%). The sensitivity and specificity of migraine were 52.6% (95% CI = 43.5-61.6%) and 78.1% (95% CI = 61.2-89.0%), respectively.

Conclusions

Approximately half of the patients had not received any specific diagnosis, and fewer than half had diagnoses consistent between headache specialists and previous physicians. These findings highlight the challenges of headache diagnosis and underscore the need for further investigation to enhance diagnostic accuracy.

## Introduction

Primary headaches are defined as headaches without underlying medical conditions that cause headaches. Studies have shown its high prevalence, estimating that 46% of adults worldwide experience headache disorders, including 11% with migraines, 42% with tension-type headaches (TTHs), and 3% with chronic daily headaches [1]. To reduce symptom intensity, frequency, and overall burden, the accurate and timely diagnosis of headaches is essential, leading to the provision of appropriate treatment, including acute and preventive medications. Misdiagnosis or delayed diagnosis can lead to inadequate management of pain and may contribute to the development of medication overuse headache (MOH) and progression to chronic headache disorders, both of which are difficult to treat [2,3].

According to the International Classification of Headache Disorders, third edition (ICHD-3), primary headaches are classified into the following four groups: migraines, TTHs, trigeminal autonomic cephalalgias (TACs), and other primary headache disorders [4]. Migraine, in particular, presents a significant challenge because of its substantial socioeconomic impact and potential to evolve into chronic migraine, a condition that often necessitates more complex and intensive treatments [3,5]. Although clinical guidelines such as the ICHD-3 and various diagnostic tools are available to support accurate diagnosis [4,6,7], challenges and delays in diagnosing headache disorders have been reported worldwide [8-14].

In Japan, primary headaches are common and impose a significant burden on individuals and society, including economic loss from absenteeism and presenteeism [15-18]. However, studies on the misdiagnosis and underdiagnosis of primary headaches in Japan are limited. Prior research in Japan has shown that only 11.6% of individuals with migraine were correctly diagnosed as having migraine and that patients with cluster headaches, a type of TAC, waited an average of 7.3 years before receiving an accurate diagnosis [19,20]. It is also reported that over one in four patients received an unconfirmed diagnosis during their initial visit [21]. A better understanding of the current state of primary headache misdiagnosis is essential for improving diagnostic accuracy, reducing delays, and addressing underdiagnosis. Therefore, among patients who were referred to headache specialists, this study examined the accuracy rate of primary headache diagnosis in Japan by comparing diagnoses made by physicians at medical institutions that patients had previously visited with those made by headache specialists as ground truth.

## Materials and methods

Study setting and population

This retrospective, observational study was conducted at the Headache Center of Tominaga Hospital in Osaka, Japan. The center is one of the country’s most renowned facilities for headache care, with seven board-certified headache physicians. The study population included patients who had consulted physicians at other institutions, including clinics, general hospitals, and university hospitals, within six months of their visit to the Headache Center. We collected diagnostic data from patients aged six years or older who visited between August 2024 and April 2025 (hereafter, the study period). Using a structured questionnaire developed by ourselves (Appendix), we collected data on patient age, sex, diagnoses made by headache specialists at the center, diagnoses made by previous physicians, and characteristics of the previous medical institution. Based on the responses from the collected questionnaires, data were entered using double-entry to ensure reliability. Only patients with diagnostic information available from both the headache specialist and the previous physician were included in the analysis. The study was approved by the Hedgehog MedTech Ethics Committee (approval number: 202401). All participants provided informed consent.

Outcome variables

The primary outcome was the accuracy rate of the previous physicians’ headache diagnoses, calculated as the consistency between their diagnoses and those of the headache specialists, assuming the latter to be correct and defining the latter as ground truth. The previous physicians’ diagnoses were determined from either referral letters or patient-provided information during the initial consultation. Diagnoses from referral letters were prioritized when patient-provided information was different from referral letters. Headache specialists were not blinded when they diagnosed patients because reading referral letters is clinically necessary to make diagnoses.

To assess diagnostic consistency, we categorized headache diagnoses into the following five groups according to ICHD-3 codes: (1) migraines; (2) TTHs; (3) TACs; (4) other primary headaches; and (5-14) other headaches, including secondary headaches, painful cranial neuropathies, and other facial pain. Free-text responses for diagnostic names were classified according to ICHD-3. To calculate the accuracy rate when multiple headache categories coexisted, migraine, TTH, TACs, other primary headaches, and other headaches were prioritized in that order. If the previous physician’s referral letter did not specify a diagnosis within the five categories (e.g., “headache”), we classified the case as undiagnosed.

Statistical analysis

First, we summarized the characteristics of patients and previous medical institutions. Institution characteristics included institution type (clinic, general hospital, or university hospital) and department of specialties that they advocate (internal medicine, neurology, neurosurgery, headache, pain management, or other). Second, we assessed the overall accuracy of the previous physicians’ headache diagnoses by comparing them with those of the headache specialists. We calculated the accuracy rate as the number of patients with consistent diagnoses across the two groups divided by the total number of patients. We also calculated the kappa coefficient by excluding patients who were not given any specific diagnoses by previous physicians. Third, we calculated accuracy, sensitivity, specificity, and positive predictive value (PPV) for each diagnostic category. Analyses were performed using Stata version 18 (StataCorp, College Station, TX, USA).

Secondary analysis

First, as a sensitivity analysis, we performed the same analysis after double-counting the patients with two or more types of headaches, considering the possibility of misclassification for them. Second, we conducted multivariable logistic regression, adjusting for patient characteristics, to identify the characteristics of prior medical institutions associated with incorrect diagnoses. The outcome variable was whether the previous physician’s diagnosis matched that of the specialist or not. Patient characteristics included sex, age (<30, 30-49, and ≥50 years), the headache specialist’s diagnosis (migraines, TTHs, TACs, or others), and the presence of combined headaches (defined as having two or more types). Given the small sample size, we combined other primary headaches and other headaches into one category (“others”). Previous medical institution characteristics included institution type (clinic and hospital) and department (specialist, internal medicine, and other). For institution type, we grouped general hospitals and university hospitals as “hospitals,” and for department, we grouped neurosurgery, neurology, pain management, and headache departments as “specialists.” Patients with missing data on institution type or department were excluded from the analysis, and there were no missing data except for institution type or department. Two-tailed p-values <0.05 were considered to indicate significance.

Subgroup analyses

To better understand headache diagnostic accuracy among nonspecialists, we examined the extent to which diagnoses varied across medical departments. Some physicians, such as neurologists and neurosurgeons, are considered to be skilled in managing headaches. Therefore, we excluded patients referred from neurology, neurosurgery, headache, and pain management departments and ran the same analyses in the rest of the physicians, supposed to be nonspecialists, to calculate the diagnostic accuracy rate.

## Results

Characteristics of the study sample and previous medical institutions

We collected the data of 152 patients, and five patients were excluded from the analysis because the diagnostic data provided by previous physicians were missing. We analyzed 147 patients (mean age = 41.5 years; SD = 18.0 years), and of these patients, 100 (68.0%) were female, and 47 (32.0%) were male (Table 1). Most patients were referred from clinics (94 patients, 64.0%), followed by general hospitals (35 patients, 23.8%) and university hospitals (5 patients, 3.4%). The most common department of the previous medical institutions was internal medicine (34 patients, 23.1%), followed by neurosurgery (22 patients, 15.0%), neurology (13 patients, 8.8%), and headache (10 patients, 6.8%). Other referring departments were ophthalmology, otolaryngology, psychiatry, obstetrics and gynecology, dermatology, pediatrics, orthopedics, dentistry, surgery, and gastroenterology.

**Table 1:** Patient characteristics and the characteristics of previous medical institutions. ^a^: Values are presented as n (%), except for age. ^b^: Previous medical institutions’ characteristics are based on information obtained from the referral letter or the patients. ^c^: Others included ophthalmology, otolaryngology, psychiatry, obstetrics and gynecology, dermatology, pediatrics, orthopedics, dentistry, surgery, and gastroenterology.

Patient characteristics		N (%)
Number of of patients		147
Age, mean (SD), years		41.5 (18.0)
Sex	Female	100 (68.0)
	Male	47 (32.0)
Previous medical institution characteristics^b^		
Type	Clinic	94 (64.0)
	General hospital	35 (23.8)
	University hospital	5 (3.4)
	Unknown	13 (8.8)
Department	Internal medicine	34 (23.1)
	Neurology	13 (8.8)
	Neurosurgery	22 (15.0)
	Headache	10 (6.8)
	Pain management	2 (1.4)
	Others^c^	34 (23.1)
	Unknown	32 (21.8)

Consistency of headache diagnosis

Of the 147 patients, 114 (77.6%) were diagnosed with migraines by headache specialists, 17 (11.6%) with TTHs, 3 (2.0%) with TACs, 7 (4.8%) with other primary headaches, and 5 (3.4%) with other headaches. One patient (0.7%) had an unconfirmed diagnosis at the initial visit (Table 2) and was excluded from further analyses. There were 14 (9.5%) patients diagnosed as having two or more headache types, including eight patients with combined migraine and TTH.

**Table 2:** Consistency of the previous physician’s headache diagnosis and headache specialist’s diagnosis. TTH: tension-type headache; TACs: trigeminal autonomic cephalalgias; CI: confidence interval

	Diagnosis made by headache specialists						Indicators of each headache category			
Diagnosis made by previous physicians	Migraine	TTH	TACs	Other primary headaches	Other headaches	Total	Accuracy rate (%) (95% CI)	Sensitivity (%) (95% CI)	Specificity (%) (95% CI)	Positive predictive value (%)
Undiagnosed	50	12	1	2	4	69	-	-	-	-
Migraine	60	4	0	2	1	67	58.2 (50.1-65.9)	52.6 (43.5-61.6)	78.1 (61.2-89.0)	89.6
TTH	4	1	0	0	0	5	86.3 (79.8-91.0)	5.9 (1.0-27.0)	96.9 (92.3-98.8)	20.0
TACs	0	0	2	0	0	2	99.3 (96.2-99.9)	66.7 (20.8-93.9)	100.0 (97.4-100.0)	100.0
Other primary headaches	0	0	0	1	0	1	95.9 (91.3-98.1)	14.3 (2.6-51.3)	100.0 (97.3-100.0)	100.0
Other headaches	0	0	0	2	0	2	95.2 (90.4-97.7)	0.0 (0.0-43.4)	95.6 (95.0-99.6)	0
Total	114	17	3	7	5	146	43.8 (36.0-51.9)	-	-	-

Among the 146 patients with definitive diagnoses by headache specialists, 69 (47.3%) had not received a specific diagnosis at previous medical institutions. The overall accuracy rate of the previous physicians’ diagnoses was 43.8% (95% confidence interval (CI) = 36.0-51.9%), assuming the headache specialists’ diagnoses were correct. The overall kappa was 0.38 when calculated by excluding 69 patients who did not receive specific diagnoses from previous physicians. The accuracy rate and specificity for migraine were the lowest among the five headache categories (58.2% and 78.1%, respectively). Among patients who received specific diagnoses at previous medical institutions, the most common misdiagnoses were migraine being mistaken for TTH and vice versa.

Secondary analysis

As a sensitivity analysis, we performed the same analysis after double-counting 14 patients with two or more types of headaches. The results were similar, as the overall accuracy rate of the previous physicians’ diagnoses was 40.6% (95% CI = 33.3-48.4%), and the overall kappa was 0.31 (Table 3). The accuracy rate and specificity for migraines were the lowest among the five headache categories (58.1% and 71.7%, respectively).

**Table 3:** Consistency of the previous physician’s headache diagnosis and headache specialist’s diagnosis with dual category retention. TTH: tension-type headache; TACs: trigeminal autonomic cephalalgias; CI: confidence interval

	Diagnosis made by headache specialists						Indicators of each headache category			
Diagnosis made by previous physicians	Migraine	TTH	TACs	Other primary headaches	Other headaches	Total	Accuracy rate (%) (95% CI)	Sensitivity (%) (95% CI)	Specificity (%) (95% CI)	Positive predictive value (%)
Undiagnosed	50	17	1	3	5	76	-	-	-	-
Migraine	60	5	0	2	6	73	58.1 (50.4-65.5)	52.6 (43.5-61.6)	71.7 (57.5-82.7)	82.2
TTH	4	2	0	0	0	6	83.8 (77.3-88.7)	8.3 (2.3-25.8)	97.1 (92.7-98.9)	33.3
TACs	0	0	2	0	0	2	99.4 (96.5-99.9)	66.7 (20.8-93.9)	100.0 (97.6-100.0)	100.0
Other primary headaches	0	0	0	1	0	1	95.6 (91.2-97.9)	12.5 (2.2-47.1)	100.0 (97.5-100.0)	100.0
Other headaches	0	0	0	2	0	2	91.9 (86.6-95.2)	0..0 (0.0-25.9)	98.7 (95.2-99.6)	0
Total	114	24	3	8	11	160	40.6 (33.3-48.4)	-	-	-

**Figure 1:**
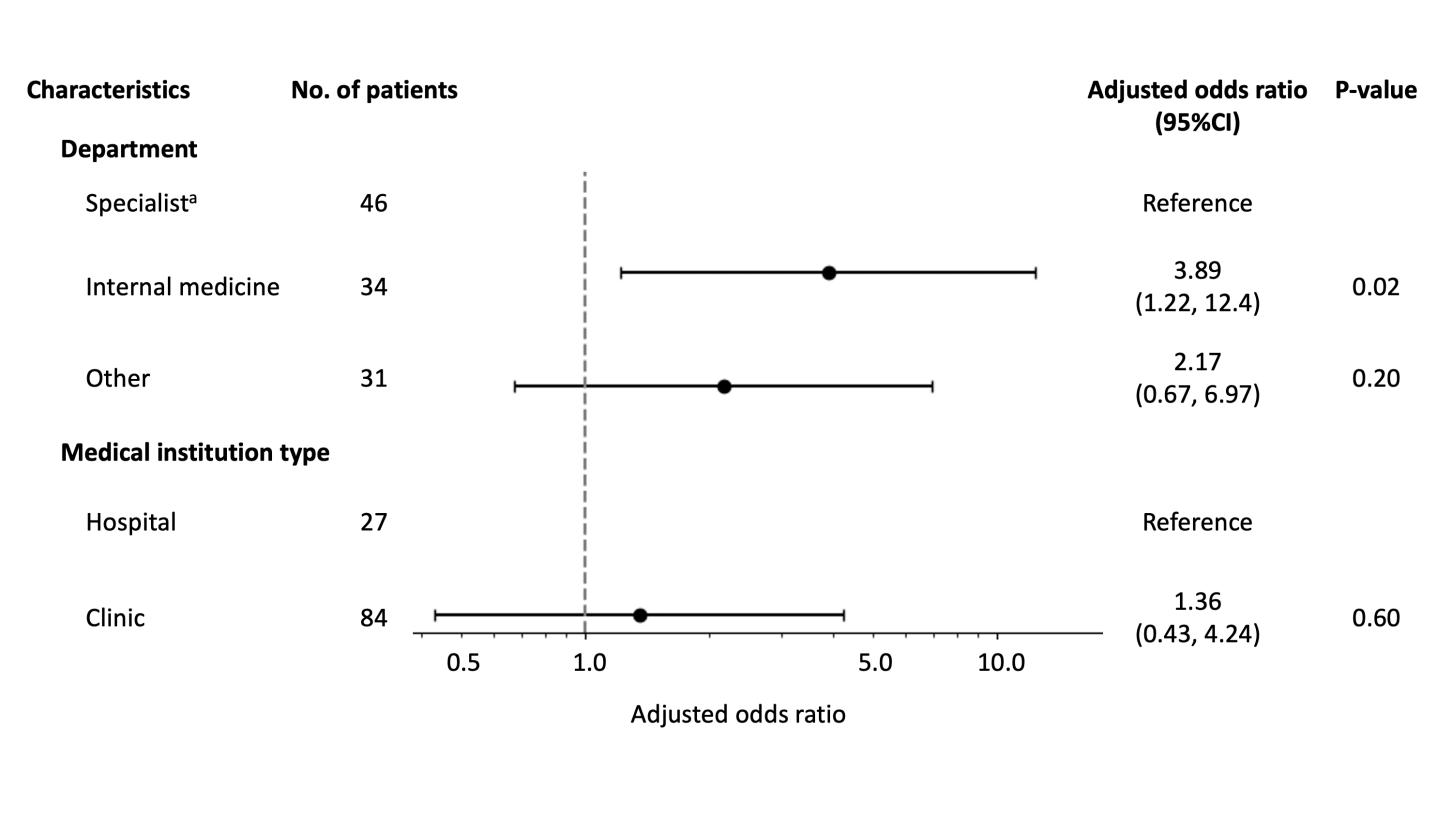
Association between characteristics of medical institutions and correct headache diagnoses. ^a^: Specialist department included neurology, neurosurgery, pain management, and headache. We examined the association between characteristics of medical institutions and correct diagnoses by using multivariable logistic regression models adjusted for patient characteristics. CI: confidence interval

After excluding patients with missing data on the type of medical institution or department (N = 36), 111 patients remained to be analyzed. Although the sample size was small, in the multivariable model adjusted for potential confounders, the diagnoses made by physicians in the internal medicine department were less concordant with those made by headache specialists (adjusted odds ratio (aOR) = 3.89; 95% confidence interval (CI) = 1.22 to 12.4) (Figure 1). We found no evidence of an association between institution type and diagnostic accuracy (aOR = 1.36; 95% CI = 0.43 to 4.24).

Subgroup analysis

After excluding patients referred from neurology, neurosurgery, pain management, and headache departments (N = 48), 99 patients remained to be analyzed. Of these, 59 (59.6%) patients were undiagnosed at previous medical institutions (Table 4). The most common definitive diagnosis by headache specialists was migraines (79 patients, 79.8%), followed by TTHs (13 patients, 13.1%). The accuracy rate of the previous diagnoses was 36.4% (95% CI = 27.6-46.2%), meaning 37 patients received consistent diagnoses from previous physicians and headache specialists. Similar to the results of the main analysis, the accuracy and specificity rates for migraine were the lowest among the five headache categories (53.5% and 90.0%, respectively).

**Table 4:** Consistency of the previous physician’s headache diagnosis and the headache specialist’s diagnosis when excluding patients who attended departments of neurology, neurosurgery, headache, and pain management at previous institutions. TTH: tension-type headache; TACs: trigeminal autonomic cephalalgias; CI: confidence interval

	Diagnosis made by headache specialists						Indicators of each headache category			
Diagnosis made by previous physicians	Migraine	TTH	TACs	Other primary headaches	Other headaches	Total	Accuracy rate (%) (95% CI)	Sensitivity (%) (95% CI)	Specificity (%) (95% CI)	Positive predictive value (%)
Undiagnosed	43	11	1	1	3	59	-	-	-	-
Migraine	35	1	0	1	0	37	53.5 (43.8-63.0)	44.3 (33.9-55.3)	90.0 (69.9-97.2)	94.6
TTH	1	1	0	0	0	2	86.9 (78.8-92.2)	7.7 (1.4-33.3)	98.8 (93.7-99.8)	50.0
TACs	0	0	0	0	0	0	99.0 (94.5-99.8)	0.0 (0.0-79.3)	100 (96.2-100.0)	-
Other primary headaches	0	0	0	0	0	0	97.0 (91.5-99.0)	0.0 (0.0-56.1)	100 (96.2-100.0)	-
Other headaches	0	0	0	1	0	1	96.0 (90.1-98.4)	0.0 (0.0-56.1)	99.0 (94.3-99.8)	0
Total	79	13	1	3	3	99	36.4 (27.6-46.2)	-	-	-

## Discussion

Approximately half of the patients in this study were not given any specific diagnoses in previous medical institutions, and the overall accuracy rate of previous physicians’ diagnoses was 43.8%. When excluding patients referred from neurology, neurosurgery, pain management, and headache departments in the previous institutions, the accuracy rate was lower, and only approximately one in three patients received a diagnosis consistent with that of headache specialists at the previous medical institutions. Additionally, compared with specialists department, internal medicine physicians were less likely to make concordant diagnoses with headache specialists. These findings suggest that primary care physicians tend to defer diagnosing the type of primary headaches before referring to headache specialists.

Although we could not determine the reasons for the low diagnostic concordance rate, there are some possible explanations. First, primary care physicians may find it difficult to diagnose the type of primary headache. Guidelines such as the ICHD-3 and various diagnostic tools, including screening questionnaires, are available to help physicians make correct diagnoses [4,6]. However, nonspecialists may not be familiar with their use and refer patients to headache specialists without making a specific diagnosis. The high number of patients with undiagnosed primary headaches and the low diagnostic accuracy, particularly in internal medicine, are consistent with previous studies. In a study conducted in UK primary care clinics, 70% of patients with headaches did not receive a diagnosis [14]. In another study in China, over half (56.6%) of patients with primary headaches remained undiagnosed [10]. In this study, considering the study population consisted of patients who were referred to headache specialists, these patients may have had more complex or atypical symptoms than general headache patients, making accurate diagnosis difficult in the primary care setting. Consequently, primary care physicians may have been unable to diagnose primary headaches before referral. This study was unable to collect data on patients’ detailed information, such as severity, symptoms, and comorbidities, so future research should include stratification by severity and complexity to estimate the true undiagnostic and misdiagnosis rate.

Second, primary care physicians may intentionally withhold labeling headache types to avoid misdiagnosis because an incorrect diagnosis can lead to patient confusion and undermine trust in medical care [22]. Therefore, physicians in primary care may focus primarily on excluding urgent secondary headaches while intentionally avoiding detailed classification of primary headache disorders. This triage is considered appropriate in primary care settings [23], but it may lead to delays in treatment for primary headaches. Especially among migraine patients, delayed or inappropriate treatment can lead to chronicity and MOH, which are difficult and costly to manage [24,25]. Therefore, prompt and accurate diagnosis is important for patient outcomes. This study cannot clarify the diagnostic delay, so future research is required to assess the diagnostic delay in the primary care setting.

Third, primary care physicians may intentionally omit their diagnoses from referral letters, despite having made a diagnosis, to avoid introducing diagnostic bias or preconceptions that could influence the specialist’s independent assessment. By providing clinical information without specifying a diagnosis, the primary care physician may be attempting to promote a more objective evaluation by the specialist. Therefore, our results may overestimate the true underdiagnosis, and further research is necessary to explore the process of headache diagnosis and assess the underdiagnosis of headaches in primary care settings.

Although we cannot identify the underlying causes of these findings in this study, it is important to consider that despite the high prevalence of headache patients [17], there were only 1,037 board-certified headache specialists in Japan as of March 2025 [26]. Although some complicated or atypical cases are undoubtedly essential to refer to headache specialists, the referral process may delay the initiation of appropriate treatment and potentially disadvantage patients. Given this shortage of headache specialists and the possibility of delaying treatment, it is desirable that accurate headache diagnosis be performed as much as possible within the primary care setting to ensure timely and appropriate management. The headache guideline in Japan recommends that primary care physicians diagnose primary headaches such as migraine, TTH, and cluster headache [27]. The guideline also recommends primary care physicians use screening tools, such as ID Migraine [28] and the 3-Question Headache Screen [29], for diagnosing primary headaches, but it is still unclear whether primary care physicians utilize such tools before referring to headache specialists.

Therefore, further research is needed to clarify how headache diagnoses are made in primary care settings and to determine the true rate of misdiagnosis and underdiagnosis. Deepening the understanding of current diagnostic practices may help optimize collaboration between primary care physicians and headache specialists, which may lead to improvement in patient outcomes.

Limitations

This study has several limitations. First, our results may be biased because of the small sample size, especially for less common headache types such as TACs, other primary headaches, and other headaches. Second, sampling bias may exist in the study population, as patients referred to a well-known headache hospital may present with more complex symptoms, which tend to be more difficult to diagnose. This may have led to certain characteristics being over- or underrepresented, such as the lower proportion of male patients in this study compared with that in the general headache population. Third, there are unmeasured confounding factors such as the patient’s comorbidities. Fourth, we were unable to obtain information on the board certification of previous physicians. Nevertheless, the results remained qualitatively unchanged in the subgroup analysis that excluded patients referred from neurology, neurosurgery, pain management, and headache departments at previous institutions. Fifth, our questionnaire has not been assessed for its validity and reliability. Sixth, observer bias may be present because headache specialists were not blinded and had known the previous physician’s diagnoses before they diagnosed. Finally, we were unable to assess the diagnostic process followed by both previous physicians and headache specialists, and further research is needed to determine the underlying causes of diagnostic challenges in headache care.

## Conclusions

In the present study, over 40% of patients did not receive any specific diagnosis at previous medical institutions, which may lead to a delay in providing adequate treatment. Further research is needed to investigate diagnostic challenges in primary care settings and explore potential interventions. A better understanding of current diagnostic practices will help specialists and primary care physicians collaborate smoothly, enabling patients to receive timely and accurate diagnoses, which would enhance patients’ outcomes.

## Appendices

**Table 5:** Structured questionnaire used in this study.

Questionnaire	Options
1. Confirmed diagnosis	a. Migraine without aura (MO)
	b. Migraine with aura (MA)
	c. Chronic migraine (CM)
	d. Episodic tension-type headache (ETH)
	e. Chronic tension-type headache (CTH)
	f. Episodic cluster headache (ECH)
	g. Chronic cluster headache (CCH)
	h. Medication-overuse headache (MOH)
	i. Other (please specify): __________________
2. Source of information regarding the initial diagnosis	a. A referral letter
	b. A patient
3. Previous physician’s diagnosis (according to a referral letter or from a patient )	a. Migraine
	b. Migraine without aura (MO)
	c. Migraine with aura (MA)
	d. Chronic migraine (CM)
	e. Tension-type headache (TTH)
	f. Episodic tension-type headache (ETH)
	g. Chronic tension-type headache (CTH)
	h. Medication-overuse headache (MOH)
	i. No diagnosis
	j. Non-specified headache (suspected primary headache)
	k. Headache (as a chief complaint)
	l. Other (please specify): __________________
4. Previous medical institution’s name	
5. Previous physician’s medical institution	a. A GP/Primary care clinic
	b. A community hospital
	c. A university hospital
	d. A headache clinic
	e. Other (please specify): _____________
6. Previous physician’s department	a. Internal medicine
	b. Neurology
	c. Neurosurgery
	d. Pain clinic
	e. Obstetrics and Gynecology
	f. Pediatrics
	g. Otorhinolaryngology (ENT)
	h. Ophthalmology
	i. Other (please specify): _____________
